# Identification and phylogenetic analysis of two canine coronavirus strains

**DOI:** 10.1186/s44149-021-00013-9

**Published:** 2021-07-19

**Authors:** Junji Gan, Ye Tang, Haifeng Lv, Wenbin Xiong, Xiaoyan Tian

**Affiliations:** 1 Animal Infectious Disease Laboratory, School of Veterinary Medicine, Yangzhou University, Yangzhou, China; 2 Jiangsu Co-Innovation Center for Prevention and Control of Important Animal Infectious Diseases and Zoonosis, Yangzhou University, Yangzhou, China; 3 Jiangsu Key Laboratory of Zoonosis, Yangzhou University, Yangzhou, China

**Keywords:** Canine coronavirus, Characterization, IIa subtype, Isolation, Phylogenetic analysis

## Abstract

Canine coronavirus (CCoV), a member of the genus *Alphacoronavirus*, is an enveloped, single-stranded positive-sense RNA virus that responsible for gastroenteritis in dogs. In this study, two CCoV isolates were successfully propagated from 53 CCoV-positive clinical specimens by serial passaging in A-72 cells. These two strains, CCoV JS1706 and CCoV JS1712, caused cytopathic effects in A-72 cells. The sizes of virus plaque formed by them differed in early passages. Electron microscopy revealed a large quantity of typical coronavirus particles with 80–120 nm in diameter in cell culture media and cytoplasm of infected cells, in which they appeared as inclusion bodies. RT-PCR analysis of *S* gene indicated that these two isolates were belonged to CCoV IIa subtype. Homology of RdRp, S, M and N proteins between the two strains were 100, 99.6, 99.2 and 100.0%, respectively, whereas they were 99.4–100%, 83.1–95.2%, 88.5–99.2% and 91.9–99.7% identity compared to CCoV II reference strains. Phylogenetic analysis of RdRp, S, M and N protein showed that they were closely related to CCoV II strains. These two subtype IIa isolates will be useful for evaluating the pathogenesis and evolution of CCoV and for developing diagnostic reagents and vaccines.

## Main text

Coronaviruses (CoVs) are animal and human pathogens that can cause respiratory and enteric diseases (V’Kovski et al. 2021). CoVs are divided into four genera termed *Alpha-*, *Beta-*, *Gamma-* and *Delta-coronavirus* based on phylogenetic analyses and genomic structures (Terada et al. 2019). Canine coronavirus (CCoV) is an enveloped, single-stranded positive-sense RNA virus belonging to the genus *Alphacoronavirus* and is considered as the main pathogen responsible for enteritis in dogs (Decaro and Buonavoglia 2008). CCoV genome is approximately 30 kb in length. Two large overlapping open reading frames (ORFs) are presented at the 5′-terminal genome region, namely, ORF1a and ORF1b, which encode two replicase polyproteins. A key enzyme required for both genome replication and transcription is RNA-dependent RNA polymerase (RdRp). Four kinds of structural proteins (including S, E, M and N) and several nonstructural proteins are encoded by ORFs downstream of the replicase gene (Decaro and Buonavoglia 2008; Ntafis et al. 2011). These structural proteins are essential for the assembly and infectivity of viral particle. S glycoprotein is associated with tropism, binding to cell-surface receptors, fusion and entry of the virus into cells (Timurkan et al. 2021). M and E proteins are involved in virus assembly. The nucleocapsid protein forms complexes with genomic RNA and plays a critical role in enhancing the efficiency of virus transcription, translation and assembly (Liu et al., 2021).

CCoV is divided into two different genotypes, namely, CCoV type I (CCoV I) and CCoV type II (CCoV II). The typical CCoV reference strain is CCoV II (Decaro and Buonavoglia 2008; Le Poder et al. 2013). According to amino acid sequence in the N-terminal region of S protein, CCoV II is further classified into two different subtypes, namely, CCoV IIa (typical strains) and CCoV IIb (TGEV-like strains). In the N-terminus of S protein of CCoV IIb, amino acid sequence is highly similar to that of infectious gastroenteritis virus (TGEV). The appearance of CCoV IIb may be due to the recombination of CCoV II and TGEV (Decaro et al. 2010; Licitra et al. 2014). 

Virus isolation and cell culture adaptation are crucial for better understanding about biological characteristics, disease diagnosis and prevention of CCoV. However, CCoVs are not easily propagated in cell culture (Pratelli et al. 2000), and only a few CCoV strains are available. Genetic information concerning CCoVs detected in recent years is scarce. In this study, we successfully isolated two strains of CCoV IIa that had stable cellular adaptability and high viral titers.

### Isolation of CCoV from clinical samples

From 2016 to 2019, CCoV isolations from clinical samples were conducted continuously in our laboratory. A total of 53 fecal samples from dogs infected with CCoV were collected in the animal hospital of Yangzhou University in China. From these dogs, CCoV was isolated from only two samples and named CCoV JS1706 and CCoV JS1712. Sample 1 was collected on 20 June 2017 from the feces of a 3-month-old male Labrador. Sample 2 was collected on 28 December 2017 from the feces of a 2-year-old male Welsh corgi. Neither dog had ever been vaccinated. Viral isolation was achieved at the 2nd to 3rd passage from the fecal sample. CPE (cell cytopathic effect) was characterized by cell rounding and lysis of the infected monolayer. Viral growth was confirmed by IFA (indirect immunofluorescence assay) using 2B8, a CCoV-specific monoclonal antibody. CCoV protein stained by the monoclonal antibody was distributed in cytoplasm but not nucleus. Viral titers (measured as TCID_50_: 50% tissue culture infective dose) of A-72 cells were 2 × 10^3^ TCID_50_/ mL (CCoV JS1706) and 1 × 10^6^ TCID_50_/ mL (CCoV JS1712) at the 4th passage. The two isolates were serially propagated in cell culture for more than 100 generations (data not shown). In this study, we verified that only a few strains were suitable for growth in vitro.

### Two virus isolates showed different biological characteristics

**Fig. 1:**
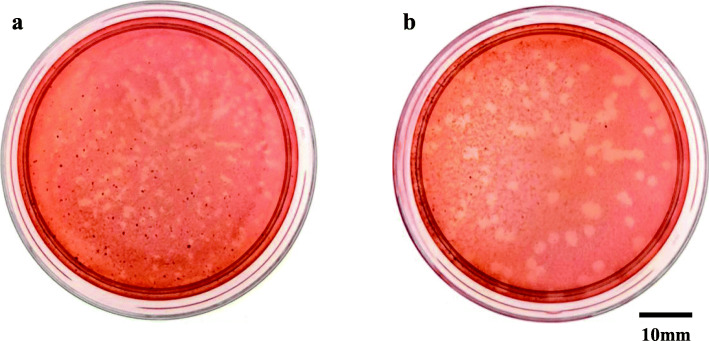
Plaque sizes of CCoV strains in A-72 cells 3 days post infection. **a**, JS1706 strain. **b**, JS1712 strain. Scale bar, 10 mm

Plaque morphology and size of the 4th generation CCoV JS1706 and CCoV JS1712 isolates are showed in Fig. 1. plaques of CCoV JS1706 strain measured 1–2 mm (Fig. 1a), while JS1712 strain produced larger uniform plaques of 3–4 mm in 3 days post infected A-72 cells (Fig. 1b). Previous studies have shown that animal coronaviruses with different plaque sizes may have different pathogenicity. For example, FIPV (feline infectious peritonitis virus) is macrophage tropic and is believed to cause aberrant cytokine and/or chemokine expression and lymphocyte depletion, resulting in lethal disease. Small plaques (1 mm) of FIPV are more virulent than the virus generated large plaques (3 mm) (Mochizuki et al. 1997). Whether the pathogenicity of CCoV JS1706 and CCoV JS1712 strains differs in vivo requires further study.

**Fig. 2:**
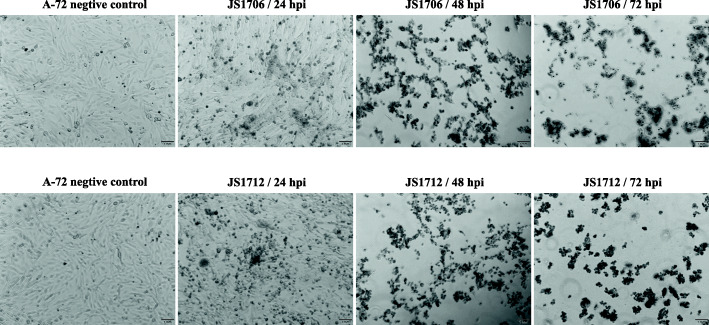
Progression of cytopathic effects caused by CCoV in A-72 cells. The cytopathic effects of A-72 cells infected with CCoV JS1706 and CCoV JS1712 were monitored under microscopy at the indicated time points. Hpi, hours post inoculation. Scale bars, 1 mm

**Fig. 3:**
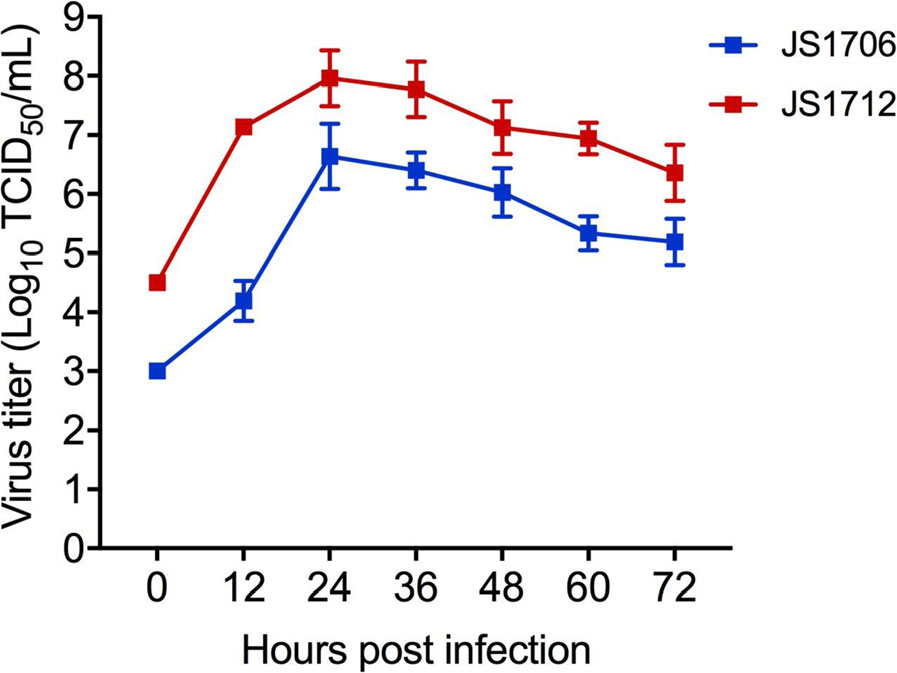
Growth kinetics of CCoV JS1706 and CCoV JS1712 in A-72 cells. A-72 cells were infected with JS1706 and JS1712 at an MOI (multiplicity of infection) of 0.05. Infected cells and culture supernatant were harvested every 12 h for 72 h. Virus titer was determined by TCID_50_ (50% tissue culture infective dose)_._ Data are representative of three independent experiments performed in triplicate and presented as the means ± SD

After CCoV JS1706 (12th passage of virus) and CCoV JS1712 (5th passage of virus) were fully adapted to cells, virus growth kinetics were evaluated using a standard infection time course. After 24 h, partial cell round-reduced CPE was observed. After 48 h of inoculation, the cytopathic rate was 80–90%, and after 72 h of inoculation, most cells detached from flask surface. CPE induced by the two strains did not significantly differed (Fig. 2). TCID_50_ determination showed that they both replicated in A-72 cells, reaching maximum titers of TCID_50_ > 10^6^/mL in 24 h post infection (Fig. 3). This result demonstrated that infected A-72 cells were able to produce CCoV rapidly.

**Fig. 4:**
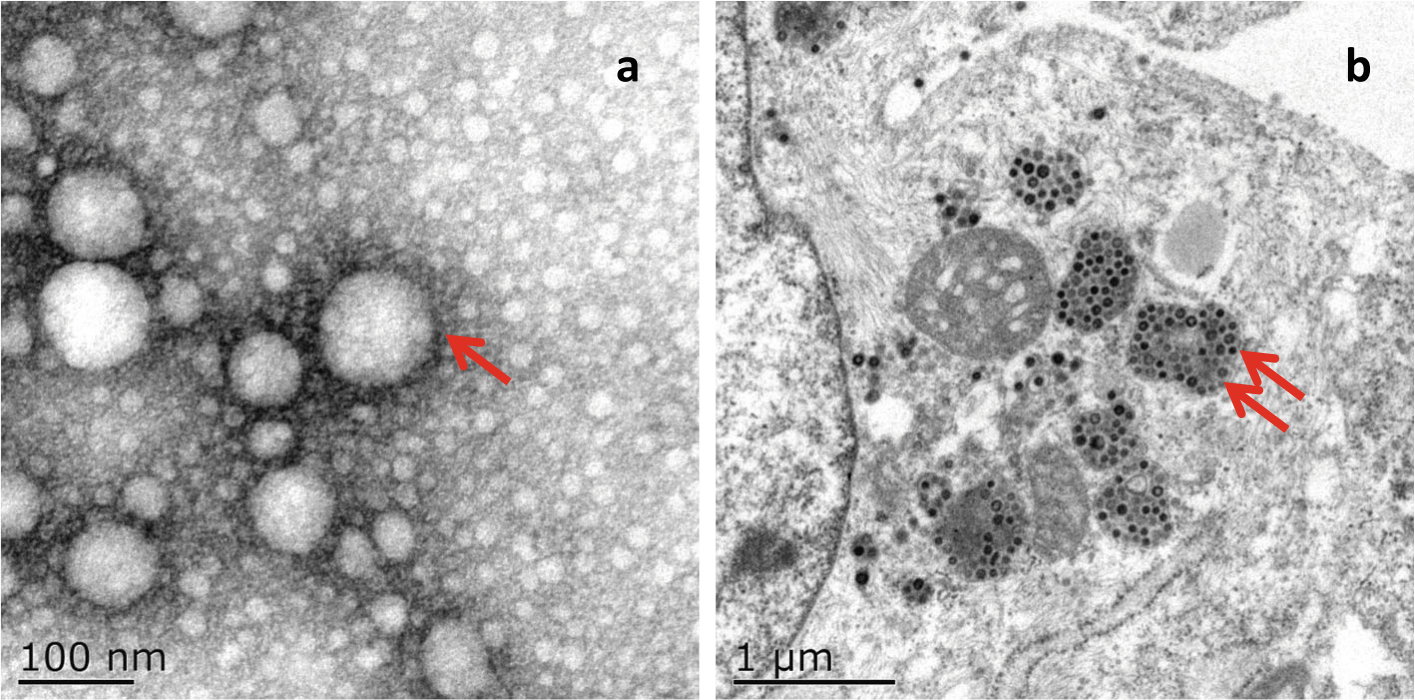
Detection of CCoV under electron microscopy. **a,** Negative staining of CCoV particles from CCoV JS1706-infected cell culture supernatant. The red arrow indicates corona spikes of CCoV particles. Scale bar, 100 nm. **b,** Ultrathin sections of A-72 cells infected with CCoV JS1712 24 h post infection. The red double arrow indicates virions present in the inclusion bodies in cytoplasm. Scale bar, 1 μm

Electron microscopy of virus supernatant showed a large number of coronavirus like particles (Fig. 4a), with most 80–120 nm in diameter. In CCoV-infected cells, multiple inclusion bodies and mature coronavirus particles were found (Fig. 4b). Virus are released by exocytosis, the secretory pathway of cell, thereby infecting new host cell.

### Two virus isolates are of the CCoV IIa subtype

A 758 bp product was amplified by using the primer pairs 20179 and NS-R-dg, which selectively recognized the CCoV IIa subtype. In addition, PCR assays with primers 20179 and 174–268 specifically for CCoV IIb subtype did not generate any amplicons. Therefore, CCoV JS1706 and CCoV JS1712 are CCoV IIa subtype viruses.

### Sequencing and phylogenetic analysis of CCoV

Sequencing results showed that the full-length *M* gene of CCoV JS1706 and CCoV JS1712 was 792 bp in length, encoding 263 amino acids (GenBank accession number: MN078152 and MN078151). The full length of *N* gene was 1149 bp, encoding 382 amino acids (GenBank accession number: MN163040 and MN163039). RdRp protein was found to be 316 amino acids in length, similar to most CCoV reference strains. *S* gene of both strains was 4362 nucleotides in length, encoding a protein of 1453 amino acids.

Sequence comparison of RdRp of two isolates revealed 100% amino acid identity and high amino acid identity (96.5–100%) with that of *Alphacoronavirus* reference strains. Amino acid sequences of S protein of the them showed 99.6% homology and 84.0–95.2% identity with reference strains of CCoV IIa and FCoV II. The complete M and N proteins of two isolates revealed 99.2 and 100% amino acid identity, respectively. They showed 88.5–99.2% and 91.9–99.7% amino acid sequence identity with CCoV II reference strains. They had lower amino acid sequence identity (88.5–89.3%) of M protein and N protein (89.1–91.9%) with CCoV II vaccine strain INSAVC. Protective efficacy of the vaccine strain against Chinese epidemic strains requires further investigation.

**Fig. 5:**
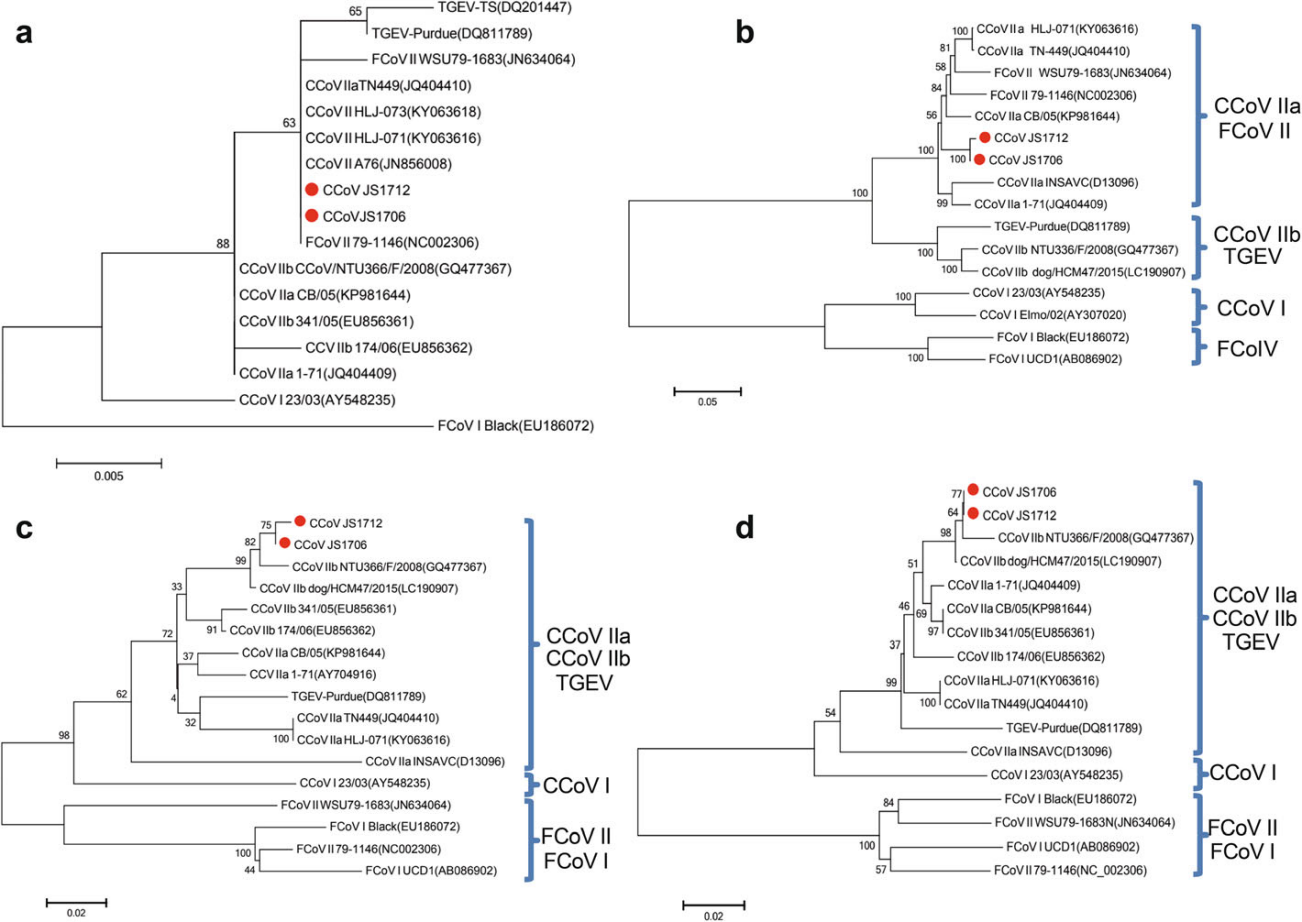
Phylogenetic analysis based on amino acid sequences of RdRp (a), S (b), M (c) and N (d) proteins. The red circles indicate strains isolated in this study. Scale bars represent the numbers of amino acid substitutions per site. The name and accession number of each strain are shown

To explore the evolution of CCoV, Four phylogenetic trees were reconstructed using RdRp, S, M and N protein (Fig. 5). Relationship of different CCoV strains was basically consistent with the results of amino acid homology analysis. In RdRp trees, various FCoV II, CCoV II and TGEV strains clustered together. Since RdRp domain is conserved across all groups, it will most likely reflect the true coronavirus phylogeny (Koçhan et al. 2021). Phylogenetic tree from the deduced amino acid sequence of S protein showed that two CCoV isolates of this research were most closely related to CCoV IIa but had a significant distance from FCoV I and CCoV I. Phylogenetic tree based on M and N proteins showed that they were closest to strain CCoV IIb NTU366/F/2008 (99.2 and 98.5%) which identified in Taiwan and CCoV IIb dog/HCM47/2015 (99.7%) which identified in Vietnam. These three strains clearly clustered into a single clade, suggesting that they may have a common ancestor. Cluster structures of M and N proteins were very similar but differed from those of S protein. In the phylogenetic tree constructed by partial S protein sequence, CCoV IIa and CCoV IIb formed two independent branches; therefore, phylogenetic analysis of S protein can provide more accurate and meaningful subtype classification.

In summary, two strains of CCoV IIa with different biological characteristics were isolated and stably propagated with high virus titers. These CCoV strains isolated in China are important tools for further studies on their pathogenesis, evolution and development of diagnostics and vaccines development.

## Methods

### Cell culture

A-72 cells (canine fibrosarcoma cell line, ATCC-CRL-1542) were used in this study. The culture medium was Dulbecco’s modified Eagle’s medium (DMEM) with 10% fetal bovine serum (FBS), penicillin (100 U/mL), and streptomycin (100 μg/mL). Maintenance medium was DMEM containing 1% FBS.

### Sampling and virus isolation

A total of 53 dogs with severe diarrhea were used for sampling. All samples were taken from dogs that were brought to the animal hospital of Yangzhou University for treatment. CCoV was tested using a rapid test kit for CCoV (BioNote Inc., Korea) and confirmed by selectively amplifying a 409 bp fragment of *M* gene as previously described (Pratelli et al.^1999^). CCoV-positive rectal swab samples were filtered and inoculated on an A-72 cell monolayer. CPE was monitored every day. Each sample was considered negative after three passages. If CPE appeared after 3–4 d of inoculation, the cells were tested for CCoV antigen by IFA using a monoclonal antibody (2B8) targeting N protein of CCoV.

### Plaque assay

Plaque size measurement and plaque purification were performed following previously described methods with some modifications (van Nguyen et al. 2017). Plaque assay was performed in 6-well plates. Confluent monolayers of A-72 cells were washed once with PBS and inoculated with 0.2 mL virus suspensions per well (diluted serially 10-fold with maintenance medium). After 90 min of incubation, the cultures were covered with 1% nutrient agar. Plates were incubated at 37 °C for 3 d and stained with 0.2% neutral red for 6 to 8 h. Diameters of plaques were measured to determine the average plaque size. Isolated CCoVs were plaque-purified three times.

### Viral growth kinetics

Viral growth kinetics of CCoV were determined as previously described (Mao et al. 2016). A-72 cells were cultured in a 6-well plate. After dense monolayer was formed in 24 h, the isolated CCoV viruses were inoculated at a MOI of 0.05 with three replicate wells. Culture plates were collected every 12 h for 3 d and stored temporarily at − 25 °C. Virus titration was performed in 96-well plates with 10-fold serial dilutions in triplicate. Inoculated cells in 96-well plates were cultured for 3 d, and CPE was monitored under a microscope. TCID_50_ was calculated according to the Reed-Muench method. The data are representative of three independent experiments.

### Electron microscopy

Negative staining and ultra-thin-section examination by electron microscopy were performed following previously described procedures (Chen et al. 2014; Pratelli et al. 2002). CCoV virus was inoculated into A-72 cells and cultured for 2–3 d. The culture was freeze-thawed and centrifuged at 4000 *g* for 30 min. Supernatant was collected and 10% (*w*/*v*) PEG6000 was added. After stirring and dissolving overnight at 4 °C, the culture was centrifuged at 4000 *g* at 4 °C for 30 min. precipitate was resuspended in TNE buffer (pH 7.4) to 1/10 of its original volume. Then, it was layered onto a 30% (*w*/*v*) sucrose-TNE cushion and ultracentrifuged at 150,000 *g* for 1.5 h. Precipitate was resuspended in TNE buffer to 1/50 of its original volume. After negative staining with 2% phosphotungstic acid, the sample was examined with a Tecnai 12 transmission electron microscope (Philips). A-72 cells infected with CCoV strain were trypsinized 24 h post infection and centrifuged at 1000 *g* for 10 min. Cell pellet was fixed with 2.5% glutaraldehyde-PBS. Ultrathin sections were prepared and stained with uranyl acetate and lead citrate and examined with an EM208 transmission electron microscope (Philips).

### Identification of CCoV subtypes

Total RNA of CCoV JS1706- and CCoV JS1712-infected cells was extracted according to the instructions of an RNA extraction kit (EasyPure RNA Kit, Transgen). The extracted RNA was stored at − 80 °C for later use. To determine the subtype of CCoV isolates, 5′-terminal of *S* gene were amplified by PCR. Primers were designed according to reports (Decaro et al. 2008; Decaro et al. 2013). For amplification CCV subtype IIa, primers 20179 (5′-GGCTCTATCACATAACTCAGTCCTAG-3′) and NS-R-dg (5′-GCTGTAACATAKTCRTCATTCCAC-3′) were used, and the expected size of amplified fragment was 758 bp. For amplification CCV subtype IIb, primers 20179 (5′-GGCTCTATCACATAACTCAGTCCTAG-3′) and 174–268 (5′-CAACATGTAACCTTTGTCTGTGATCTGC-3′) were chosen, and the expected size of amplified fragment was 499 bp. Reaction system was prepared according to the instructions of the OneStep RT-PCR kit (QIAGEN). RT-PCR conditions were as follows: reverse-transcription reaction at 50 °C for 30 min; pre-denaturation at 95 °C for 10 min; denaturation at 94 °C for 30 s, annealing at 55 °C for 30 s, extension at 72 °C for 50 s, in 35 cycles; and final extension at 72 °C for 10 min. PCR products were electrophoresed in 1% agarose (BIO-RAD, PowerPac), and the image was processed using a gel-imaging system (BIO-RAD, GelDoc XR+).

### Sequencing and phylogenetic analysis

Published primers were used to generate full-length *S*, *M* and *N* genes of CCoV isolates (Decaro et al. 2007; Ma et al. 2008). Briefly, three partially overlapping fragments encompassing *S* gene were amplified using primer pairs El-Ins1/S2, SIIF/SR2 and SF3/SR3. MF (5′-CATATAACCCTGATGAAGCA-3′) and MR (5′-GGCCACGAGAATTGGAACGAC-3′) were used to amplify *M* gene. The expected size of amplified fragment was 918 bp. NF (5′-CTAAAGCTGGTGATTACTCAACAG-3′) and NR (5′-TAATAAATACAGCGTGGAGGAAAAC-3′) were used to amplify *N* gene. The size of amplified products was 1272 bp. Primers amplifying RdRp (979 bp) were designed based on the available canine coronavirus sequence (CB/05, KP981644), Nsp12F (AGTGCTGGTTATCCTTTGAACAAGT) and Nsp12R (AGTCTCCAGGTCCAACAA). RT-PCR was performed according to the instructions of the OneStep RT-PCR kit. PCR products were electrophoresed on a 1% agarose gel and subjected to sequence analysis (Sangon Biotech Company, Shanghai, China).

Sequence assembly and analysis were carried out using DNAStar 7.1/MegAlign software package. Phylogenetic analysis was conducted using MEGA6 program. Phylogenetic trees based on amino acid sequences of RdRp, S, M and N proteins were generated using the neighbor-joining method. Bootstrap values were calculated based on 1,000 replicates. Reference sequences of CCoV, TGEV and feline coronaviruses (FCoVs) belonging to the same genera were obtained from GenBank and used for sequence alignment and phylogenetic analysis.

## Data Availability

The datasets used and analyzed during the current study are available from the corresponding author on reasonable request.

## Abbreviations

CCoV: canine coronavirus
FCoV: feline coronavirus
TGEV: transmissible gastroenteritis virus
EM: electron microscopy
DMEM: Dulbecco’s modified Eagle medium
FBS: fetal bovine serum
TCID_50_: 50% tissue culture infective dose
ORFs: open reading frames
IFA: indirect immunofluorescence assay
MOI: multiplicity of infection
